# When Policy Exists but Practice Fails: Rethinking Cognitive Screening as a Public Health Delivery Problem

**DOI:** 10.5888/pcd23.260152

**Published:** 2026-07-16

**Authors:** Brian Kim

**Affiliations:** 1 Auburn University College of Sciences and Mathematics, Auburn, Alabama

The first time I administered a validated cognitive screening tool to an older adult in a rural Alabama senior center, she said, “No [student] doctor has ever asked me these questions.” I was an undergraduate volunteer, Montreal Cognitive Assessment (MoCA)–certified but not a clinician, and her remark captured a larger pattern. The structured cognitive assessment she was receiving from a trained student had never been offered to her by the primary care physician she had seen for more than a decade, despite Medicare policy requiring cognitive assessment during Medicare annual wellness visits (1). Her experience is not unusual, particularly in rural communities, where early cognitive impairment often goes undetected in routine primary care and where older adults face the fewest alternative routes to evaluation.

This essay proposes that the persistent gap in early dementia detection in the US is primarily a public health delivery problem rather than a gap in knowledge, policy, or screening tools, and that this delivery gap disproportionately affects rural older adults. Medicare requires cognitive assessment during the Medicare annual wellness visit, and validated instruments like the MoCA have existed for 2 decades (2). Yet cognitive impairment continues to be identified late, and this delay is most pronounced in rural communities where clinic-based delivery intersects with transportation barriers, specialist scarcity, and reduced primary care access. The question, then, is how and where screening is delivered, and for whom current models are working.

A Government Accountability Office analysis of Medicare claims from 2018 through 2022 found that only 2.4% of traditional Medicare beneficiaries with an Alzheimer diagnosis had received the dedicated cognitive assessment and care planning service, and that urban beneficiaries used this service at roughly twice the rate of rural beneficiaries (3). Although utilization tripled during the study, absolute uptake remained low, and the 2020 through 2021 portion of this period coincided with documented nationwide disruptions in COVID-19–related preventive care. Even accounting for that disruption, the persistent rural-urban gap and low overall uptake point to a structural delivery problem that predates and outlasts the pandemic. In routine practice, cognitive assessment during the Medicare annual wellness visit is often omitted, rushed, or reduced to informal approaches that lack sensitivity for detecting mild cognitive impairment (4). When screening is suboptimal, early cognitive changes may be missed, delaying early interventions while neurodegenerative processes continue.

These delivery gaps reflect predictable pressures on any health care system managing simultaneous workforce constraints and growing demographic demand. Primary care clinicians face limited visit time, competing preventive priorities, and uncertainty about how to respond to abnormal screening results, particularly in rural settings where referral pathways to neurology and geriatrics are scarce (3). At the patient level, fear of diagnosis, stigma surrounding dementia, and normalization of memory loss as aging discourage older adults from raising concerns. These system- and patient-level factors create a self-reinforcing cycle in which cognitive impairment progresses until crisis forces recognition.

These factors are magnified in rural communities, where preventive services are delivered almost exclusively through clinic-based models. Rural older adults may face social isolation and transportation barriers, along with reduced access to primary care and specialty services (5). Many no longer drive and rely on family members who may be unavailable during clinic hours. Where public transit does operate in rural counties, it is typically restricted to weekday daytime hours, requires advance scheduling, and serves only designated corridors, and a substantial minority of US counties have no rural transit service at all (6). When cognitive screening depends solely on clinic attendance, screening becomes practically inaccessible to the populations at greatest risk. Rural older adults could be largely excluded from preventive screening by a delivery system structured around clinic access rather than population reach.

Working in church fellowship halls, senior centers, and public housing common rooms across East Alabama, I have encountered older adults with long-standing memory concerns who had never received formal cognitive screening. Many had a primary care physician, but reaching a clinic-based evaluation, whether during a Medicare annual wellness visit or through specialist referral, required reliable transportation, scheduling flexibility, and confidence navigating a system many perceived as intimidating. These observations informed a student-led, community-based memory screening initiative built on a simple principle: if older adults cannot reliably reach clinic-based screening, screening must be brought to where they already are. Trained, MoCA-certified students administer validated assessments in community centers, churches, and housing facilities, shifting tasks to extend population-level preventive reach (7). Across multiple rural counties, this initiative has screened and identified approximately 145 older adults whose results warranted formal cognitive evaluation and follow-up, and each received a standardized cognitive health summary to share with their primary care provider. Whether these recommendations translate into completed clinical evaluation depends on the downstream capacity of the surrounding health system.

The credibility of a student-led model depends on documented training, defined scope of practice, and institutional anchoring. Every screener completes the publisher-endorsed MoCA certification program before administering the instrument, standardizing scoring and administration across sites. Certification is supplemented by program-specific orientation on common confounders of cognitive screening, including sensory impairment, acute illness, depression, and low baseline literacy, together with scripted procedures for informed verbal consent and disclosure of results. The program operates under faculty advisors from Auburn University’s School of Nursing and Harrison College of Pharmacy and uses a peer supervision model in which newer screeners are trained and observed by more experienced student leaders, consistent with the task-shifting framework underlying delivery of community-based preventive services (7). Students do not provide diagnostic interpretations; however, any screen at or below threshold generates a cognitive health summary designed to support rather than replace formal clinical evaluation.

This student-led model is one of several emerging approaches that extend detection beyond the primary care clinic. In Wisconsin, a state-supported initiative embedded within 25 aging and disability resource centers has delivered community-based screening in 39 counties and 5 tribal communities, using existing aging services infrastructure and routing positive screens back to primary care (8). In Georgia, the state-funded Georgia Memory Net operates specialized memory assessment clinics paired with community services educators, addressing the downstream gap that forms when rural clinicians identify cognitive concerns but lack an accessible specialty resource (9). Together with student-led screening in Alabama, these models illustrate that a broader pattern, demonstrating progress on early detection, depends on infrastructure that sits outside the traditional clinic visit, whether anchored in aging services, specialty referral networks, or trained nonspecialist personnel. Each focused on a different barrier in the delivery chain, and the approaches are complementary. Federal frameworks have increasingly focused on the development, expansion, and empowerment of the community health care workforce and infrastructure, including emerging federal investments in dementia-capable community health worker programs (10) and the Centers for Medicare and Medicaid Services’ Guiding an Improved Dementia Experience (GUIDE) Model (11). The collaboration and synergy between federal, state, and local efforts could have a broader impact on early screening and detection of cognitive impairments among older adults.

A complementary policy approach would be to strengthen the Medicare annual wellness visit itself by requiring specific validated cognitive screening instruments rather than leaving tool selection to clinician discretion. This would improve screening consistency among older adults who attend Medicare annual wellness visits but cannot reach those least likely to attend. Mandating better tools during the visit would increase screening quality for those who reach the clinic; community-based screening would decrease missed opportunities by reaching older adults who rarely attend clinic-based preventive visits.

Two legitimate concerns deserve direct acknowledgment. The first is false-positive MoCA results, which can generate anxiety and consume clinical resources. This student-led initiative mitigates that risk through the standardized cognitive health summary, which documents the result, the instrument used, and specific follow-up guidance, ensuring a structured handoff to primary care. The second concern is downstream capacity. Rural communities have persistent shortages of neurologists, geriatricians, and neuropsychologists, and even willing primary care clinicians often lack clear pathways for formal cognitive workup. Identifying individuals for follow-up does not by itself guarantee that evaluation, diagnosis, or treatment will occur. This limitation reinforces the case for pairing community detection with investment in rural dementia care infrastructure, including specialty referral networks like Georgia Memory Net (9) and emerging dementia-capable community health worker programs (10).

Dementia care in the US remains anchored to clinic-based detection despite evidence that this model might underserve older rural adults. The models in Wisconsin, Georgia, and Alabama differ in personnel, setting, and entry point, yet share 3 features that account for their reach: they operate in trusted community settings outside the primary care clinic, they use standardized protocols that connect detection or diagnosis back to primary care, and they depend on stable institutional sponsorship. A scalable national model would combine state-level public health sponsorship, partnerships with existing community infrastructure, and dedicated funding streams that sustain community-based screening and specialty referral as recognized public health services. Federal frameworks provide the policy support; what they lack is consistent investment in community-level infrastructure and coordination. The tools exist, the mandates exist, and working models exist. The remaining task is to treat community-based detection as core public health infrastructure, funded and sustained accordingly, so that early cognitive screening reaches the rural and underserved populations that current delivery systems continue to miss. The collaboration among federal, state, and local efforts can bring more early screening and detection of cognitive impairments among older adults, and therefore, bring better health outcomes.
